# Supplementary figures and images for: A CRISPR-Cas12a-based universal rapid scrub typhus diagnostic method targeting 16S rRNA of Orientia tsutsugamushi
Source: PLoS Negl Trop Dis. 2025 Jan 22;19(1):e0012826. doi: 10.1371/journal.pntd.0012826 (PMC11790230; doi:10.1371/journal.pntd.0012826)

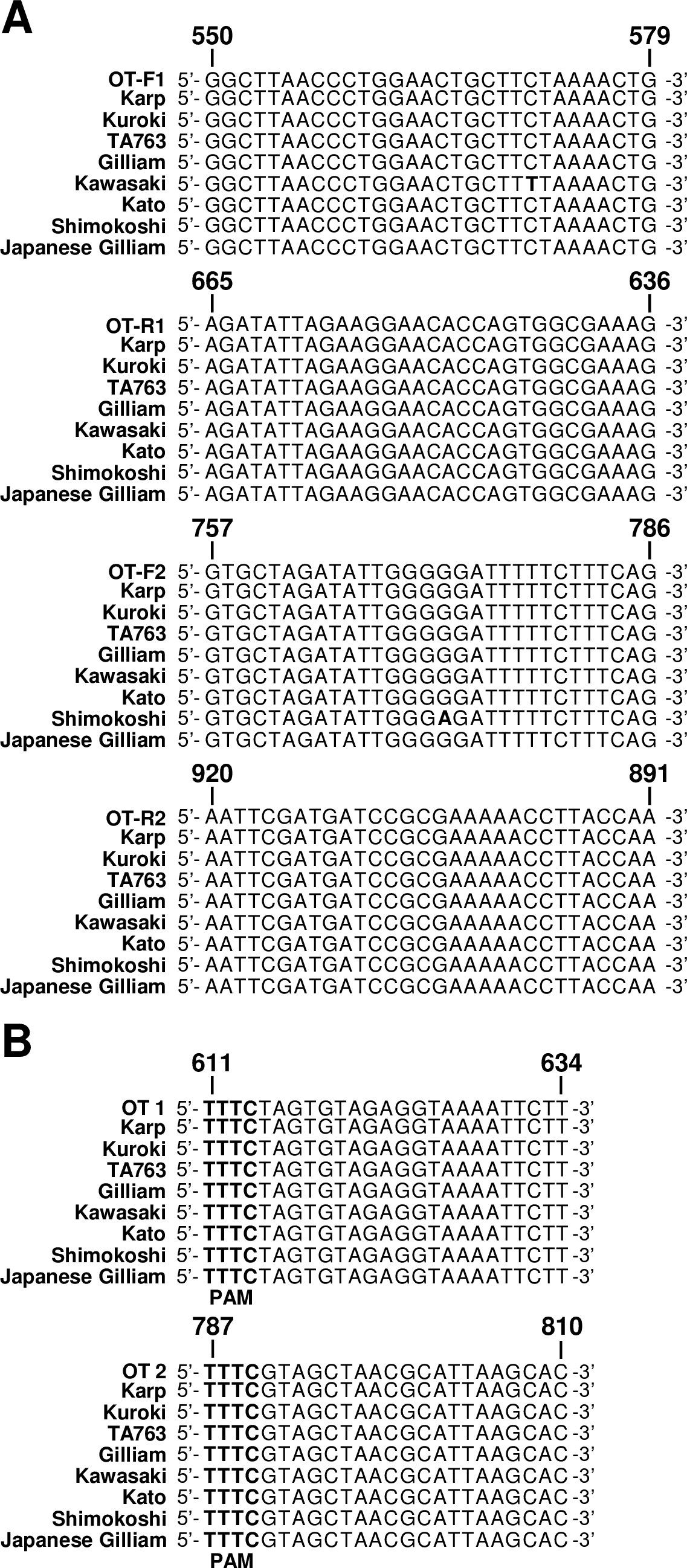

Supplement: S1 Fig — Sequence alignment of the (A) RPA primers and (B) gRNAs used in this study, relative to the different genotypes of O. tsutsugamushi. Sequences of OT1-R and OT2-R are shown in the reverse-complement orientation. (TIF) [file pntd.0012826.s001.tif]

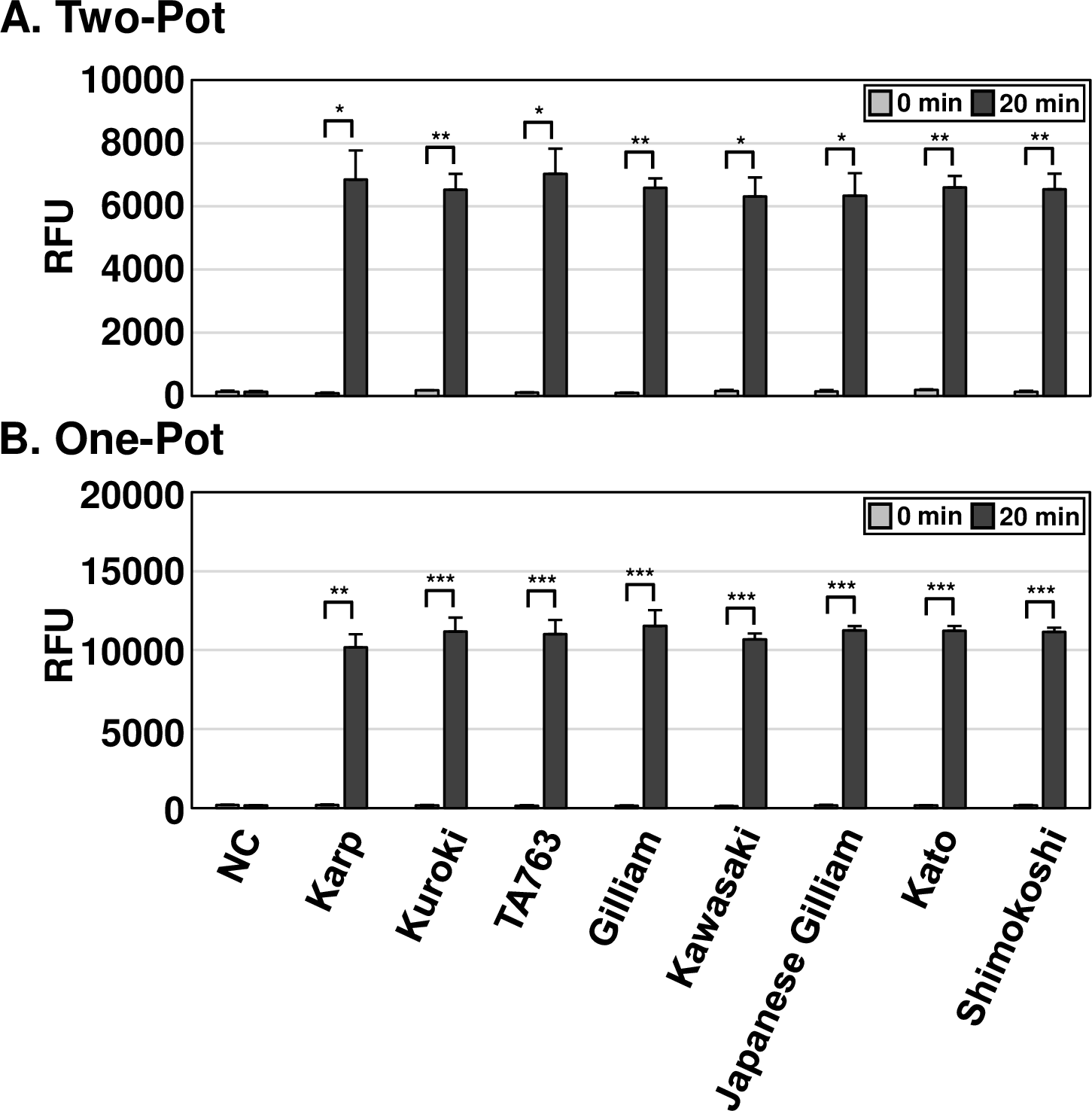

Supplement: S2 Fig — The in vitro transcribed RNA fragments from the 16S rRNA sequences of major O. tsutsugamushi strains (Karp, Kuroki, TA763, Gilliam, Kawasaki, Japanese Gilliam, Kato, and Shimokoshi) were used to evalulate (A) Two-pot DETECTR and (B) One-pot DETECTR. Values are presented as means ± s.d. (error bars) (n = 3 replicates; *** P < 0.001, ** P < 0.01, * P < 0.05 between samples, two-sample t-test). RFU, relative fluorescence unit; NC, no template control. (TIF) [file pntd.0012826.s002.tif]

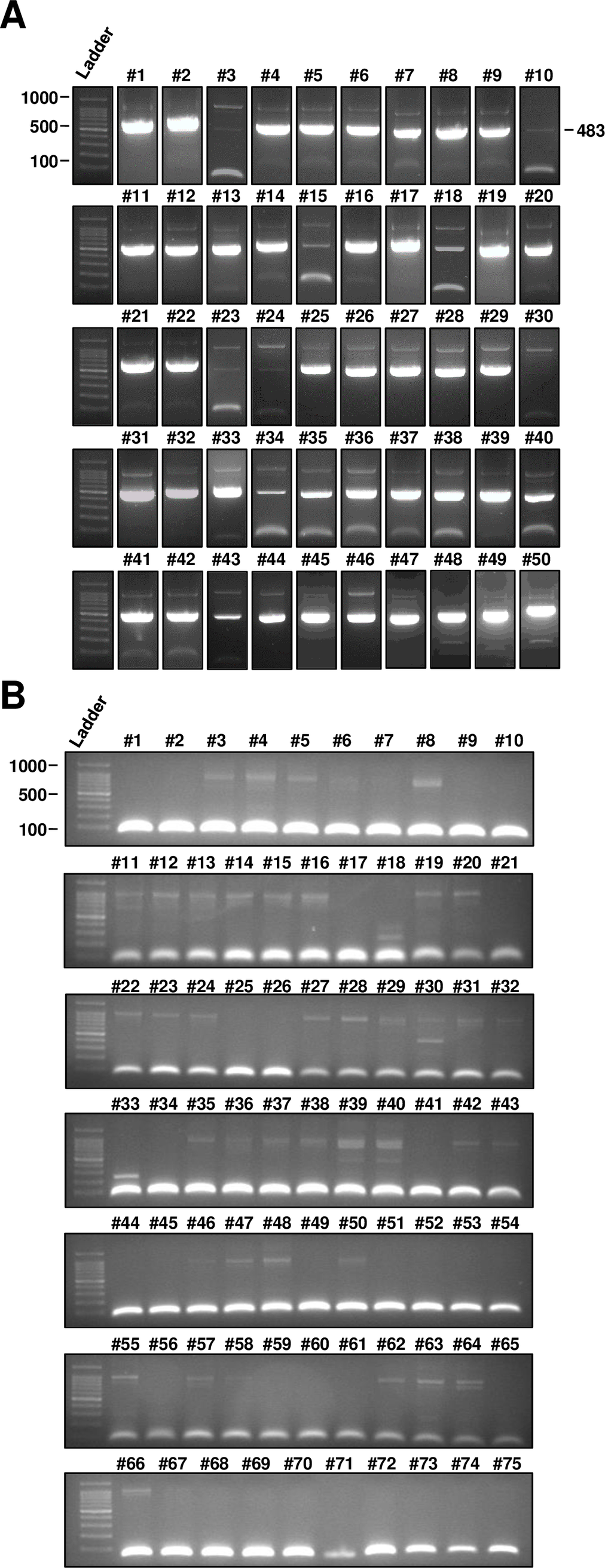

Supplement: S3 Fig — Nested PCR was performed to assess O. tsutsugamushi infection in 125 clinical samples from patients believed to be positive or negative for infection, and the product (483 bp) was visualized via gel electrophoresis. Of the total 125 samples, (A) 50 samples were confirmed positive for O. tsutsugamushi infection, and (B) 75 samples were confirmed negative for O. tsutsugamushi infection. (TIF) [file pntd.0012826.s003.tif]

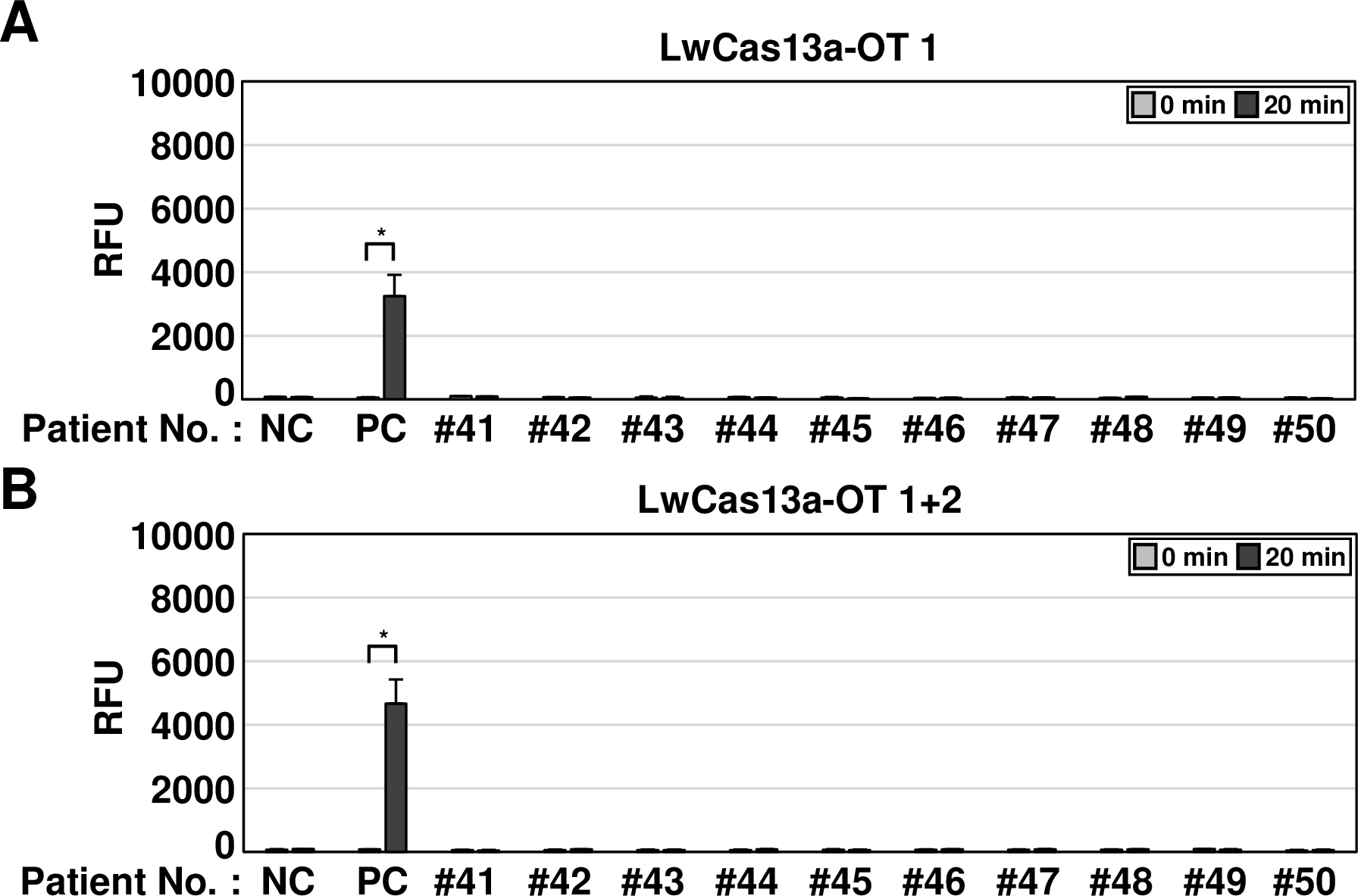

Supplement: S4 Fig — Clinical samples from 10 patients with confirmed O. tsutsugamushi infection were analyzed using OT DETECTR with (A) Cas13a-OT1 gRNA or (B) multiple gRNAs (Cas13a-OT1 and Cas13a-OT2). Values are presented as means ± s.d. (error bars) (n = 3 replicates; * p < 0.05 between samples, two-sample t-test). RFU, relative fluorescence unit; NC, no template control; PC, Positive control (in vitro transcribed O. tsutsugamushi 16S rRNA) (TIF) [file pntd.0012826.s004.tif]
