# Supplementary material for: A CRISPR-Cas12a-based universal rapid scrub typhus diagnostic method targeting 16S rRNA of Orientia tsutsugamushi
Source: PLoS Negl Trop Dis. 2025 Jan 22;19(1):e0012826. doi: 10.1371/journal.pntd.0012826 (PMC11790230; doi:10.1371/journal.pntd.0012826)
Supplement: S1 Table — (DOCX) [file pntd.0012826.s005.docx]

**S1 Table. 16S rRNAs of *Rickettsia* spp.**

| **Group** | **NCBI Accession No.** | **Organisms** | **Synthesized sequences** |
| --- | --- | --- | --- |
| Typhus | NR_044656.2 | *Rickettsia prowazekii* | 5’ – GGCTTAACCTCGGAATTGCTTTCAAAACTACTAATCTAGAGTGTAGTAGGGGATGATGGAATTCCTAGTGTAGAGGTGAAATTCTTAGATATTAGGAGGAACACCGGTGGCGAAGG  - 3’ |
|  | NR_036948.1 | *Rickettsia typhi* |  |
| Spotted fever | L36107.1 | *Rickettsia conorii* |  |
|  | NR_036848.1 | *Rickettsia sibirica* |  |
|  | NR_028018.1 | *Rickettsia rickettsii* |  |
|  | L36098.1 | *Rickettsia africae* |  |
|  | NR_036773.1 | *Rickettsia australis* |  |
|  | NR_029154.1 | *Rickettsia akari* |  |
|  | NR_179179.1 | *Rickettsia slovaca* |  |
|  | DQ365810.1 | *Rickettsia raoultii* |  |
|  | NR_115686.1 | *Rickettsia monacensis* |  |
|  | NR_026042.1 | *Rickettsia aeschlimannii* |  |
|  | NR_025919.1 | *Rickettsia massiliae* |  |
|  | NR_029156.1 | *Rickettsia parkeri* |  |
|  | NR_074459.2 | *Rickettsia japonica* |  |
|  | AF178037.2 | *Rickettsia heilongjiangensis* |  |
|  | OM912382.1 | *Rickettsia felis* |  |
|  | LC388765.1 | *Rickettsia helvetica* |  |
