# Supplementary material for: A CRISPR-Cas12a-based universal rapid scrub typhus diagnostic method targeting 16S rRNA of Orientia tsutsugamushi
Source: PLoS Negl Trop Dis. 2025 Jan 22;19(1):e0012826. doi: 10.1371/journal.pntd.0012826 (PMC11790230; doi:10.1371/journal.pntd.0012826)
Supplement: S2 Table — (DOCX) [file pntd.0012826.s006.docx]

**S2 Table. Information on patients with positive *O. tsutsugamushi* infection.**

| **Patient No.** | **Age** | **Sex** | **Immunosuppressed (Y/N)** | **Charlson Comorbidity Index** | **days of illness** |
| --- | --- | --- | --- | --- | --- |
| **#1** | **48** | **M** | **N** | **0** | **6** |
| **#2** | **72** | **M** | **N** | **3** | **5** |
| **#3** | **53** | **M** | **N** | **1** | **3** |
| **#4** | **71** | **F** | **N** | **3** | **7** |
| **#5** | **84** | **F** | **N** | **4** | **2** |
| **#6** | **63** | **F** | **N** | **2** | **7** |
| **#7** | **68** | **F** | **N** | **3** | **7** |
| **#8** | **N/A** | | | | |
| **#9** | **N/A** | | | | |
| **#10** | **81** | **F** | **N** | **4** | **4** |
| **#11** | **N/A** | | | | |
| **#12** | **48** | **M** | **N** | **0** | **2** |
| **#13** | **48** | **F** | **N** | **0** | **2** |
| **#14** | **N/A** | | | | |
| **#15** | **N/A** | | | | |
| **#16** | **N/A** | | | | |
| **#17** | **69** | **M** | **N** | **2** | **1** |
| **#18** | **60** | **M** | **N** | **3** | **4** |
| **#19** | **53** | **F** | **N** | **1** | **10** |
| **#20** | **70** | **M** | **N** | **3** | **7** |
| **#21** | **69** | **F** | **N** | **2** | **3** |
| **#22** | **62** | **M** | **N** | **2** | **10** |
| **#23** | **57** | **M** | **N** | **1** | **3** |
| **#24** | **57** | **M** | **N** |  | **5** |
| **#25** | **40** | **F** | **N** | **0** | **1** |
| **#26** | **43** | **F** | **N** | **0** | **5** |
| **#27** | **57** | **F** | **N** | **1** | **5** |
| **#28** | **57** | **M** | **N** | **2** | **5** |
| **#29** | **57** | **M** | **N** |  | **30** |
| **#30** | **50** | **M** | **N** | **1** | **6** |
| **#31** | **68** | **M** | **Y** | **9** | **14** |
| **#32** | **74** | **F** | **N** | **3** | **6** |
| **#33** | **71** | **F** | **N** | **4** | **7** |
| **#34** | **73** | **F** | **N** | **0** | **0** |
| **#35** | **43** | **M** | **N** | **0** | **5** |
| **#36** | **66** | **F** | **N** | **3** | **5** |
| **#37** | **57** | **F** | **N** | **2** | **8** |
| **#38** | **53** | **F** | **N** | **0** | **4** |
| **#39** | **70** | **F** | **N** | **0** | **7** |
| **#40** | **37** | **F** | **N** | **0** | **7** |
| **#41** | **57** | **M** | **Y** | **3** | **7** |
| **#42** | **57** | **M** | **Y** | **3** | **8** |
| **#43** | **61** | **M** | **N** | **0** | **5** |
| **#44** | **61** | **M** | **N** | **0** | **6** |
| **#45** | **63** | **F** | **N** | **0** | **0** |
| **#46** | **60** | **M** | **N** | **0** | **8** |
| **#47** | **76** | **F** | **N** | **0** | **3** |
| **#48** | **54** | **M** | **N** | **3** | **3** |
| **#49** | **52** | **M** | **N** | **1** | **5** |
| **#50** | **41** | **F** | **N** | **0** | **11** |

Missing data is indicated as "N/A" (Not Available) due to lack of patient consent for sharing specific information.
