# Supplementary material for: A CRISPR-Cas12a-based universal rapid scrub typhus diagnostic method targeting 16S rRNA of Orientia tsutsugamushi
Source: PLoS Negl Trop Dis. 2025 Jan 22;19(1):e0012826. doi: 10.1371/journal.pntd.0012826 (PMC11790230; doi:10.1371/journal.pntd.0012826)
Supplement: S1 Materials and Methods — (DOCX) [file pntd.0012826.s007.docx]

**SUPPLEMENTARY MATERIALS AND METHODS**

Cas13a cleavage reaction. LwCas13a from *Leptotrichia wadeii* was used for the Cas13a cleavage reaction test. As described for the OT DETECTR, two LwCas13a-specific gRNAs (Cas13a-OT1 and Cas13a-OT2, corresponding to nucleotides 605-634 and 787-816, respectively; Integrated DNA Technologies, Coralville, IA, USA) were designed to detect *O. tsutsugamushi* (Table S1). The LwCas13a cleavage reaction mixture contained 0.8 μL of 7 μM LwCas13a (MCLAB, San Francisco, CA, USA), 5 μL of 1 μM LwCas13a-specific gRNA (Integrated DNA Technologies, Coralville, IA, USA), 2 μL of 10 μM FQ-RNA reporter (/56-FAM/rUrUrUrUrU/3IABkFQ/; Integrated DNA Technologies, Coralville, IA, USA), and 2 μL RNase inhibitor (Enzynomics, Daejeon, Korea), and the RNA sample and 1X Cas13a reaction buffer were added to obtain a final volume of 100 μL. The reaction mixture was incubated at 37°C for 20 min, with fluorescence assessed at the beginning and end of this incubation period (λex, 485 nm; λem, 535 nm).
