# Supplementary material for: A CRISPR-Cas12a-based universal rapid scrub typhus diagnostic method targeting 16S rRNA of Orientia tsutsugamushi
Source: PLoS Negl Trop Dis. 2025 Jan 22;19(1):e0012826. doi: 10.1371/journal.pntd.0012826 (PMC11790230; doi:10.1371/journal.pntd.0012826)
Supplement: Attachment — Submitted filename: Response to Revierwers.pdf [file pntd.0012826.s008.pdf]

December 10, 2024

To: Editor, PLOS Neglected Tropical Diseases

Dear Editor,

Please find below our response to the reviewers' comments on the manuscript# PNTD-D-24-01350 (Title: A CRISPR-Cas12a-based universal rapid scrub typhus diagnostic method targeting 16s rRNA of Orientia tsutsugamushi) addressed item by item. We appreciate the comments of the reviewers and your assistance.

Thank you.

Best regards,

Yoon-Jae Song, Ph.D.

Professor, Department of Life Sciences, Gachon University, Seongnam-Si, Korea 13120

Email) songyj@gachon.ac.kr / Phone) 82-31-750-8731

# Response to the reviewers' comments

## Reviewer #1

### Methods:

While the objectives are generally clear, the study design and population description have limitations, particularly in terms of sample size and generalizability. The statistical analyses, though appropriate, could be more robustly presented, and no significant ethical concerns are evident. The methods section of the manuscript has several deficiencies that could affect the robustness and generalizability of the results.

**1. First, the sample size of 125 clinical samples, while providing an initial proof of concept, is too small to offer strong statistical power or broad applicability across different populations and regions. The authors should consider increasing the sample size and including a more diverse set of samples, both geographically and in terms of disease severity, to better evaluate the test's performance. Issues such as age, sex, days**

**of illness, incredibly important in diagnostic circumstances and this information should be presented in the manuscript.**

- As mentioned by the reviewer, we acknowledge that the sample size of 125 clinical samples provides an initial proof of concept but may appear limited in terms of statistical power and generalizability. The incidence of Tsutsugamushi infections in Korea is relatively low, and these infections are highly seasonal, predominantly occurring between October and December. This limited incidence and strong seasonality posed practical challenges for significantly increasing the sample size within a short time frame. The number of samples used in this study includes 50 positive samples and 75 negative samples, which meets the statistical criteria for evaluating sensitivity and specificity. Based on a confidence level of 95%, a margin of error of ±10%, and a statistical power of 90%, the minimum required sample size was calculated to be 50, and this study fully satisfies that requirement. Therefore, the sample size in this study is statistically valid and provides reliable data for assessing the performance of the diagnostic device in an initial diagnostic study.

- We have added a table (S2 Table) summarizing the patient information for positive samples. This table includes data for patients who consented to the use of their clinical information, including sex, age, immunosuppression status, Charlson Comorbidity Index (CCI), and days of illness prior to sample collection. Information from patients who did not consent has been excluded to adhere to ethical standards (lines 345-348)

**2. Additionally, the lack of field-based testing limits the study's ability to address its objective of developing a point-of-care diagnostic tool. Testing under real-world conditions in resource-limited settings would provide critical insights into the feasibility and robustness of the test in practical applications.**

- The reason for the absence of field testing in the current study is to comply with the requirements set forth by the Ministry of Food and Drug Safety (MFDS) in Korea for *in vitro* diagnostic medical devices (IVD) clinical trials and field testing. Currently, our study is focused on evaluating the diagnostic accuracy and reproducibility of the method within a laboratory environment, serving as a critical foundation for future KFDA (Korea Food and Drug Administration) approval. Field testing will proceed after regulatory approval, which will further establish the reliability and generalizability of the diagnostic method through validation in real-world conditions. Plans for field testing of the diagnostic kit have been added to the discussion section (lines 421-424). We also provided additional remarks on the limitation of OT DETECTR (400-407, 405-437).

**3. Moreover, the statistical analysis, while appropriate in the use of Student's t-test, lacks detailed presentation of critical values such as confidence intervals and p-values. The authors should enhance their statistical analysis by including these details and ensuring that significance is clearly marked in their results, especially within figures**

**and tables. This would strengthen the reliability of the findings and allow for a clearer interpretation of the data.**

**-** Thank you for your suggestion regarding the statistical analysis. In response, we have revised the figures to indicate significance with an asterisk (*) for cases where $P < 0.05$. (Figures 3, 4, 5 and 6; Supplementary figures 2 and 4). The t-test results for each sample were not provided separately due to the extensive volume of data; instead, we opted to directly mark statistical significance on the figures to focus on delivering the most critical information. Additionally, as the diagnostic method determines positivity based on the increase in fluorescence values, validation through *P*-values alone is sufficient, and confidence intervals were deemed unnecessary. While we acknowledge the usefulness of confidence intervals in many contexts, their exclusion in this study does not compromise the interpretive clarity or statistical validity of the results.

**Results:**

**1. I would consider revising the statistical analysis. While the manuscript mentions the use of Student's t-test to evaluate differences between groups, the results lack thorough presentation of statistical significance directly within the figures and text. Important statistical details, such as confidence intervals and exact p-values, are not consistently provided, which weakens the impact of the findings. In some figures (e.g., Figures 2, 5, and 6), statistically significant differences are not marked, making it difficult for readers to quickly assess the reliability of the data.**

- To effectively convey key statistical information, statistically significant results have been clearly marked with asterisks (*) (Figures 3, 4, 5 and 6; S2and S4 figures)**.**

**-** The primary objective of Figure 2 is to evaluate the limit of detection (LOD) of OT DETECTR, conducted with reference to regulatory guidelines such as CLSI EP17-A2. This experiment was designed to determine whether the assay consistently detects the target at specific concentrations (Detection/Non-detection) rather than to compare statistical differences between groups. This approach is widely used for LOD determination, focusing on establishing the sensitivity threshold of the assay rather than analyzing statistical differences among data points. As such, statistical measures like *P*-values or confidence intervals are not applicable in this context. Instead, the LOD was determined as the lowest concentration consistently detected in all six replicates. The revised manuscript has been updated to more clearly convey the purpose and analytical context of Figure 2 (lines 280-282).

**2. What is the point of the sensitivity comparisons? The sensitivity comparison between DNA and RNA targets (e.g., in Figure 2) could be more clearly explained. While it is mentioned that RNA-based detection is more sensitive, the reasons for this improved sensitivity are not fully explored, and the implications for clinical applications are not**

**discussed in sufficient detail. This omission leaves readers without a clear understanding of the practical significance of these findings.**

- The purpose of this comparison is to evaluate the potential of RNA-based detection of *O. tsutsugamushi* to provide higher sensitivity compared to DNA-based detection. As confirmed through the rrnDB database, *O. tsutsugamushi* has only a single copy of the 16S rRNA gene (DNA). Furthermore, previous studies have shown that transcribed 16S rRNA is present in high concentrations within the bacterium, making it an effective detection target (lines 107-110). In Figure 2, we observed that RNA-based detection demonstrated higher sensitivity than DNA-based detection. Higher sensitivity of RNA-based detection may also be attributed to the abundant transcription of 16S rRNA (lines 377-381). To address this further, we have expanded on the theoretical basis, experimental validation, and clinical applicability of RNA-based detection in the revised manuscript (lines 125-128, 377-381).

**3.The results for clinical samples, presented in Table 1, lack a breakdown of potential variability in patient presentations or sample quality. Issues such as days of illness and days of presentation are not considered – please provide this information in the revision of the manuscript.**

- To address variability in patient conditions, we have added a S2 Table summarizing the information on patients with positive *O. tsutsugamushi* infection such as age, sex, immunosuppression status, the Charlson Comorbidity Index (CCI) and days of illness.

**4. The manuscript does not explore whether the one false negative result was related to sample handling, disease stage, or other clinical factors, which limits the interpretation of the test's real-world applicability.**

- We have revised the manuscript to discuss the false-negative result in lines 391-394.

**5. The figures and tables in the manuscript could benefit from some enhancements to improve their clarity and accessibility. Specifically, Figure 2, which compares the sensitivity of the OT DETECTR to Orientia tsutsugamushi DNA and RNA, has small labels and data points that are difficult to read. Increasing the font size of axes labels and improving the color contrast between data points would enhance its legibility. Similarly, Figures 5 and 6, which present the clinical applicability of the two-pot and one-pot DETECTR assays, could be improved by adding annotations to indicate statistically significant differences directly on the graphs, making the results more immediately clear.**

- Figures 2, 5 and 6 have been revised as suggested by the reviewer.

**Conclusions:**

**1. The discussion and general conclusion in the manuscript present a promising outlook on the CRISPR-Cas12a-based diagnostic tool for scrub typhus, but the limitations are not sufficiently detailed or critically examined. While the authors emphasize the potential for rapid detection and field applicability, they do not adequately address key challenges such as the test's reliance on RNA extraction, which is difficult to manage in low-resource settings. Additionally, the limitations of the small sample size and lack of field testing are underplayed, which could affect the generalizability of the findings to real-world conditions. Furthermore, while the discussion briefly acknowledges the need for improved nucleic acid extraction and portability, the authors should have provided a more detailed exploration of how these technological barriers might impact the test's practicality in resource-limited settings. The conclusion, though optimistic, could be more balanced by discussing these limitations in greater depth and suggesting clear pathways for overcoming them, such as developing more robust, simplified sample processing methods or conducting larger field-based trials. Addressing these gaps would make the conclusion more grounded and credible.**

- As suggested by the reviewer, the manuscript has been revised to discuss the limitations of OT DETECTR (lines: 400-407, 425-437).

**Editorial and Data Presentation Modifications?**
**While the manuscript is generally well-written, focusing on improving sentence clarity, streamlining some repetitive sections, and ensuring consistent formatting will improve its editorial quality. Proofreading for minor grammatical issues and enhancing the flow in certain areas will make it more reader-friendly.**

**1. It is unclear why the tables are clearly out of order - please re-order them so they read sequentially.**
- As suggested by the reviewer, the tables have been re-ordered.

**2. Please provide page numbers in the next revision.**

- We have included page numbers in the revised manuscript.

**Summary and General Comments:**
**1. While the CRISPR-Cas12a-based OT DETECTR method shows potential in a controlled laboratory environment, its application in real-world clinical and field settings faces several challenges. These include lower sensitivity compared to nested PCR, technical difficulties related to RNA extraction and handling, and the need for further validation in diverse clinical samples. To be viable as a widely adopted**

**diagnostic tool for scrub typhus, these issues must be addressed, particularly to ensure its reliability in resource-limited environments where the disease is prevalent.**

- As addressed above, the manuscript has been revised to cover the reviewer's concerns.

## Reviewer #2

**Summary and General Comments**:

**The manuscript presents a CRISPR-Cas12a-based diagnostic method for detecting Orientia tsutsugamushi, the causative agent of scrub typhus. The work is interesting and provides a valuable tool for rapid diagnosis.**

**Major Comments:**
**1. Logical Structure of the Introduction: The introduction lacks a clear flow of ideas. It directly delves into the biological details of O. tsutsugamushi without first establishing the background and the need for rapid diagnosis. I recommend reorganizing the introduction to first address the clinical significance of scrub typhus and the challenges posed by current diagnostic methods. Then, introduce the biological characteristics of the pathogen and explain why 16S rRNA was chosen as the detection target. The current discussion of antigen variability does not provide sufficient justification for the research focus of this work.**
- Following the reviewer's suggestion, we have revised the introduction section (lines 61-137).

**2. Overemphasis on Historical Background: The historical context of scrub typhus occupies a significant portion of the introduction, without directly contributing to the study's objectives. I suggest simplifying or removing much of the historical background to better maintain focus on the diagnostic challenges and the novel aspects of this study.**
- The historical background has been removed from the manuscript.

**3. Specificity Testing: The specificity validation in the manuscript is not comprehensive, as no tests are included against pathogens closely related to O. tsutsugamushi. To strengthen the reliability of the specificity data, I recommend including additional tests with related bacterial species. This would support the claim of high specificity and reduce potential concerns about cross-reactivity.**

**-** In response to the reviewer's comment, we conducted additional specificity testing on *Rickettsia* spp., which is closely related to *O. tsutsugamushi*. Details of the *Rickettsia* spp. tested and the experimental results have been included in the manuscript (lines 230-235, 313-319, 324-327, 330, Table 2 and Figure 4).

## Reviewer #3

**Methods:**

**1. The methods section is well-written, and the objectives are clear and appropriate. It would be helpful to specify the instrumentation and software used for fluorescence measurement in the assay.**

- In response to the reviewer's comment, details regarding the fluorescence reader and software version have been included in the manuscript (lines 179-181).

**2. Additionally, could you elaborate on the rationale behind selecting specific strains like S. aureus and K. pneumoniae for specificity testing? Why were other Rickettsia species or other vector-borne pathogens not included?-**

**--** *Staphylococcus aureus* (SA), *Klebsiella pneumoniae* (KP), *Salmonella enteritidis* (SE), and *E. coli* were included in the specificity testing as they are common clinical pathogens that may co-occur with *O. tsutsugamushi*, posing a risk of misidentification. This inclusion allowed us to validate the assay's specificity and confirm the absence of cross-reactivity with these frequently encountered pathogens in similar clinical settings.

**-** In response to the reviewer's comment, we conducted additional specificity testing on *Rickettsia* spp., which is closely related to *O. tsutsugamushi*. Details of the *Rickettsia* spp. tested and the experimental results have been included in the manuscript (lines 230-235, Table 2 and Figure 4).

**Results:**

**1. Patient #27 Analysis: Could you expand on the potential reasons for missing one patient (Patient #27)? Was there something unique about this case, such as a low bacterial load, that could impact the sensitivity of the assay?**

- For Patient #27, the assay result showed a ratio of 0.5, significantly lower than the ratios exceeding 1.6 observed in other samples. This suggests that low sample purity may have impacted assay performance, potentially reducing sensitivity. Impurities or inhibitors in the sample could interfere with amplification and detection efficiency. This observation is discussed in the manuscript to underscore the influence of sample quality on assay sensitivity. Additionally, we acknowledge the need to optimize sample preparation to ensure consistent performance across diverse sample conditions (lines 391-394)

**Conclusions:**

**1. The Discussion does a great job summarizing the study's key findings, comparing OT DETECTR to existing methods, and outlining future directions for diagnostic development. However, it would be beneficial to address the limitations of OT DETECTR in this section.**

**-** In response to the reviewer's comment, we have revised the discussion section to address the limitations of OT DETECTR (lines 400-407, 425-437)

**Editorial and Data Presentation Modifications:**

**1.Line 48-49: Change "kDA" to "kDa."**

- Changed as suggested by the reviewer (lines 95-96)

**Summary and General Comments**

**1. The authors have developed a CRISPR-Cas12a-based diagnostic method to detect multiple genotypes of O. tsutsugamushi by targeting the bacterial 16S rRNA. This method has been widely utilized for pathogen diagnosis, particularly due to its low cost, minimal instrument requirements, and reduced need for technical expertise. The OT DETECTR assay will be particularly valuable in field and low-resource laboratory settings, as it demonstrates high sensitivity and specificity. The authors provide a thorough and clear background on Orientia tsutsugamushi. However, it would be beneficial to briefly elaborate on how antigenic variability impacts diagnosis and why this diversity complicates traditional diagnostic methods. This addition could strengthen the rationale for the use of molecular diagnostics in this context.**

- Frequent genetic recombination in the major surface protein TSA56 reduces the sensitivity and specificity of traditional serological diagnostic methods, as they often fail to detect diverse strains effectively. To address this limitation, we developed a molecular diagnostic method targeting the highly conserved 16S rRNA gene. This approach mitigates the challenges posed by antigenic variability, enabling reliable detection across a wide range of strains. The Introduction section has been revised to discuss how antigenic variability complicates traditional diagnostics and to highlight the advantages of the molecular strategy proposed in this study (lines 130-134)

## Reviewer #4

**Methods:**

**1. The term "universal diagnosis" should be validated with additional strains of Orientia spp. to ensure broader applicability. or it should demonstrate a board range of detection across known O. tsutsugamushi genotypes in the manuscript.**

- In response, we synthesized plasmid DNA for major strains with available sequence data from NCBI and produced RNA via *in vitro* transcription. The assay was tested for its ability to detect these strains, yielding successful detection for all tested major strains and demonstrating the diagnostic method's broad applicability (S2 Fig.). These findings have been incorporated into the Results section, with detailed information about the major strains provided in the Materials and Methods section (lines 225-235 and 319-322 and S2 Fig).

**2.In the study design you mentioned "We designed primer-gRNA sets to detect nine genotypes of O. tsutsugamushi". Have you validated the detection efficacy for all nine genotypes in real samples?**

- We consider the detection of the 8 major *O. tsutsugamushi* strains in an *in vitro* environment sufficient to validate the assay's specificity and range at this stage. Testing with real samples containing these strains is unnecessary, as the *in vitro* results already confirm the assay's ability to detect diverse genotypes (S2 Fig).

**3.For test validation, it would be beneficial to explain why Staphylococcus aureus (SA), Klebsiella pneumoniae (KP), Salmonella enteritidis (SE), and E. coli were used. Additionally, consider including more closely related bacteria from the Rickettsia group to strengthen the validation. If not please address why they were not include?**

-- *Staphylococcus aureus* (SA), *Klebsiella pneumoniae* (KP), *Salmonella enteritidis* (SE), and *E. coli* were included in the specificity testing as they are common clinical pathogens that may co-occur with *O. tsutsugamushi*, posing a risk of misidentification. This inclusion allowed us to validate the assay's specificity and confirm the absence of cross-reactivity with these frequently encountered pathogens in similar clinical settings.

**-** In response to the reviewer's comment, we conducted additional specificity testing on *Rickettsia* spp., which is closely related to *O. tsutsugamushi*. Details of the *Rickettsia* spp. tested and the experimental results have been included in the manuscript (lines 230-235, 313-319, 324-327, 312, Table 2 and Figure 4).

**4.Do you plan to include a positive control or quality control for the each test runs? or provide explanation how will you validate each run.**

- In each experiment, we used *O. tsutsugamushi* genomic RNA (100 ng) as a positive control, although it was not initially included in the presented results. The figures have now been revised to incorporate the positive control for each test run, ensuring clarity and validation of the assay (Fig. 5 and 6).

**5.Consider conducting a concordance analysis for positive predictive agreement (PPA) or negative productive agreement (NPA) with the reference assay.**

- A table presenting the concordance analysis between the conventional diagnostic method (nested PCR) and OT DETECTR has been added (Table 4)

**6.In this study, the two assays used for validation are 'the OT DETECTR' targeting 16S rRNA and nested PCR targeting the 56 kDa gene. Please discuss this issue further, especially in light of the findings that there are 10-fold differences in the sensitivity.**

- The reason why nested-PCR exhibits 10 times higher sensitivity than DETECTR is its two-step amplification process, which effectively amplifies even trace amounts of target DNA. In nested-PCR, the product generated from the first amplification is used as the template for the second amplification, significantly improving its ability to detect target DNA even at very low initial concentrations. This dual amplification process allows nested-PCR to achieve 10 times higher sensitivity than DETECTR for low-copy-number genomes. However, compared to the long reaction time of 3 hours 45 minutes for nested-PCR, OT DETECTR utilizes isothermal amplification and completes within 1 hour, making it a more suitable diagnostic technology for scrub typhus, where rapid diagnosis is critical. We have revised the manuscript to discuss this issue (lines 384-388).

**7.Line 223: "All O. tsutsugamushi cases were laboratory confirmed at Jeju National University Hospital (Jeju, Korea)". Please provide more detail on the laboratory confirmation process. For clinical samples, scrub typhus infection is typically confirmed using a combination of antibody detection (such as serological assays; IFA) and antigen detection methods (e.g., PCR or Nested PCR) or bacterial isolation.**

- Jeju National University Hospital utilized nested PCR to detect *O. tsutsugamushi* genes and confirm infection in clinical samples. This reliable diagnostic method, specifically designed for *O. tsutsugamushi*, has been appropriately reflected in the revised manuscript (lines 244-245).

**8.Line 227: Nested PCR was performed as previously described to detect the 56-kDa antigen of O. tsutsugamushi in extracted bacterial DNA. Nested PCR not detect 56-kDa antigen, but detect the gene encoding the 56-kDa antigen, please clarify/correct.**

- The typographical error on line 249 has been corrected.

**9.Line 275: (B) OT2. (n = 6 replicates). No "." After OT2**

- Changed as suggested by the reviewer (line 294)

**Results:**

**1. In the plots, for Y axis labeled "Fluorescence" please specify the units of detection.**

- The labeling has been changed to relative fluorescence unit (RFU).

**2.In the plots, for X axis suggest RNA copies/reaction.**

- The labeling for X axis has been changed as suggested by the reviewer.

**3.Do you have information on the O. tsutsugamushi genotypes in confirmed scrub typhus cases? Do you have a plan to perform genotyping on these samples?**

- Thank you for your inquiry regarding the genotyping of *O. tsutsugamushi* and our future research plans. Currently, we have positive clinical samples confirmed as the Taguchi genotype (a subtype of Kawasaki) and the Boryong genotype (a subtype of Kuroki). These samples were successfully detected by our assay, demonstrating its effectiveness across these genotypes. As this study focuses on developing and validating a diagnostic method for *O. tsutsugamushi*, we do not plan to conduct additional genotyping. We hope this clarifies the scope and contributions of our research.

**4. Have you compared this newly developed assay with quantitative detection methods (such as qPCR assay in unit of copies/ml)? If not, please consider discussing how this comparison could enhance its applicability in clinical cases.**

- To address this, we have included a discussion comparing the two methods, emphasizing the qualitative and rapid detection focus of OT DETECTR versus the quantitative capabilities of qPCR. Additionally, we have outlined plans for future studies to directly compare OT DETECTR and qPCR to further assess its analytical performance and clinical applicability. These updates are included in the revised Discussion section (lines 400-407)

**5.Have you statistically compare the fluorescent intensity obtained from two-Pot versus one-Pot approach?**

- The Cas12a diagnostic method is a qualitative approach designed to distinguish between positive and negative results. As such, statistical comparison of fluorescence intensities was deemed unnecessary. Both methods showed consistent results in terms of the limit of

detection (LoD) and positive/negative outcomes for actual samples, suggesting that direct comparison of fluorescence values does not add meaningful validation to the diagnostic method.

**6.Could you provide details on demographic background and/or clinical sings of the scrub typhus confirmed cases used in the study?**

- In response to the reviewer's comment, we have added a table (S2 Table) summarizing the patient information for positive samples. This table includes data for patients who consented to the use of their clinical information, including sex, age, immunosuppression status, Charlson Comorbidity Index (CCI), and days of illness prior to sample collection (lines 345-348). Information from patients who did not consent has been excluded to adhere to ethical standards.

**7.Line 314: "nested PCR" is a gold-standard test for diagnosing scrub typhus. Do you have any reference for the use of nested PCR as a gold standard for diagnosing scrub typhus.**

- We acknowledge that nested PCR is not a gold-standard test. The manuscript has been revised to indicate that it is a commonly used method for detecting *O. tsutsugamushi* in Korea (line 336).

**8.Line 348: Italic for O. tsutsugamushi**

- Changed as suggested by the reviewer (line 338)

**Conclusions:**

**1.Using the phase "Data not shown" can reduce transparency of the discussion suggest to avoid data not shown statement in the manuscript.**

- The data have been included in the manuscript as S4 Figure.

**2.Please consider discussing the cost evaluation of the CRISPR-Cas12a-based POCT in comparison to the currently used diagnostic processes in hospitals**

- As this study focuses on the technical validation of the diagnostic method, a detailed cost analysis was not conducted. Currently, the pricing of OT DETECTR reflects pre-commercial, research-phase costs, which are likely to change significantly with large-scale production and commercialization. Therefore, providing an accurate cost comparison with existing hospital diagnostic methods is not feasible at this stage. We acknowledge the importance of

evaluating cost efficiency and plan to perform a comprehensive cost analysis in future studies once OT DETECTR advances to commercialization. This will be critical in demonstrating its applicability, especially in resource-limited settings.

**Summary and General Comments**

**1. This manuscript is overall well-written and presents a promising new method that could be a valuable tool for scrub typhus diagnostics. However, there are areas that could benefit from improvement as mentioned above.**

- The manuscript has been revised as suggested by the reviewer.
